# The Role of the Mylohyoid Line in the Spread of Mandibular Odontogenic Deep Neck Infection

**DOI:** 10.1111/odi.70149

**Published:** 2025-11-19

**Authors:** Eiji Iwata, Kyoichi Obata, Shogo Kikuta, Naoki Kaneko, Kotaro Sato, Norio Kitagawa, Yohei Takeshita, Katsuhisa Matsuo, Junsei Sameshima, Akira Tachibana, Shintaro Kawano, Jingo Kusukawa, Masaya Akashi, Soichiro Ibaragi, Joe Iwanaga

**Affiliations:** ^1^ Department of Oral and Maxillofacial Surgery, Graduate School of Medicine, Dentistry and Pharmaceutical Sciences Okayama University Okayama Japan; ^2^ Department of Oral and Maxillofacial Surgery Kakogawa Central City Hospital Kakogawa Japan; ^3^ Department of Oral and Maxillofacial Surgery, Kobe University Graduate School of Medicine Kobe Japan; ^4^ Dental and Oral Medical Center Kurume University School of Medicine Kurume Japan; ^5^ Section of Oral and Maxillofacial Oncology, Division of Maxillofacial Diagnostic and Surgical Sciences, Faculty of Dental Science Kyushu University Fukuoka Japan; ^6^ Department of Oral and Maxillofacial Surgery, Nagoya University Graduate School of Medicine Nagoya Japan; ^7^ Department of Oral and Maxillofacial Anatomy, Graduate School of Medical and Dental Sciences Institute of Science Tokyo Tokyo Japan; ^8^ Department of Oral and Maxillofacial Radiology, Okayama University Graduate School of Medicine, Dentistry and Pharmaceutical Sciences Okayama Japan; ^9^ Department of Neurosurgery, Tulane Center for Clinical Neurosciences Tulane University School of Medicine New Orleans Louisiana USA

**Keywords:** causative tooth, mylohyoid line, odontogenic deep neck abscesses, odontogenic deep neck infections, transmission pathway

## Abstract

**Introduction:**

Although mandibular odontogenic deep neck infections are occasionally fatal, the transmission pathway has not been elucidated.

**Materials and Methods:**

This multicenter retrospective study was comprised of the patients of both sexes who were over 18 years of age and who had mandibular odontogenic deep neck abscesses. The patients' characteristics, laboratory tests, and radiographic findings were analyzed.

**Results:**

One hundred eighteen patients with mandibular odontogenic deep neck abscesses were included. Bone resorption superior to the mylohyoid line and the related abscess formation in submandibular space or submental space were both significantly associated with the presence of sublingual space abscess. In addition, the type of causative tooth was not a risk factor for abscess formation in both the sublingual space and “submandibular or submental” space.

**Conclusions:**

When an odontogenic lesion is located superior to the mylohyoid line, the abscess tends to initially form in the sublingual space and subsequently spread to the submandibular or submental space. Since any mandibular tooth can lead to abscess formation in these regions, oral and maxillofacial surgeons should carefully assess the anatomical position of the lesion and accurately identify the causative tooth.

## 1 Introduction

Mandibular odontogenic infections can rapidly progress into deep neck infections (DNIs), threatening airway patency and vital structures due to the complex anatomy of the oral and cervical regions (Kitamura 2018; Ohshima et al. 2004). Ludwig's angina is a particularly severe form of DNI, characterized by cellulitis involving the submental, sublingual, and submandibular spaces (Saifeldeen and Evans 2004; Larsson et al. 1982; Bridwell et al. 2021; Wegrzyn and Greenberg 2024). The lack of true fascial boundaries between these spaces facilitates the rapid spread of infection, often resulting in airway emergencies (Bridwell et al. 2021; Wegrzyn and Greenberg 2024). Although modern imaging and treatment have reduced mortality rates to approximately 8% in treated cases, the rate remains as high as 50% in untreated patients (Saifeldeen and Evans 2004; Nanda et al. 2017; Moreland 1988). Early identification and intervention are therefore critical.

Traditionally, the anatomical relationship between the apex of mandibular teeth and the mylohyoid line has been used to predict the pathway of infection spread. It is commonly believed that infections from teeth with apices located superior to the mylohyoid line (e.g., premolars and first molars) tend to spread to the sublingual space, while those inferior to the line (e.g., second and third molars) spread to the submandibular space (Patel and Bhatt 2018; French et al. 2017). However, this assumption is largely based on anatomical inference rather than empirical evidence, and clinical observations often reveal inconsistencies.

The aim of this multicenter retrospective study is to clarify the transmission pathways of mandibular odontogenic deep neck abscesses, with a particular focus on the role of the mylohyoid line in determining the direction of infection spread. By analyzing patient characteristics, causative teeth, and radiographic findings, we seek to provide evidence‐based insights that can improve diagnostic accuracy and surgical decision‐making in cases of odontogenic DNIs.

## 2 Methods

### 2.1 Patients

Patient records from five institutions between January 2012 and March 2023 were reviewed, and the patients diagnosed and treated for abscess in the submental space, sublingual space, or submandibular space caused by severe odontogenic infections were extracted. The patients aged over 18 years, with abscess formation observed on contrast‐enhanced computed tomography (CT), and who were hospitalized for the treatment with drip antibiotics for over 48 h were included in this study. All examinations, including contrast‐enhanced CT and blood tests, were performed at the time of admission. The hospitalization criteria were as follows: clinical findings such as skin erythema, dysphagia, difficulty eating, and high inflammation in blood tests. Exclusion criteria included patients who underwent contrast‐enhanced CT and refused participation following the study's publication.

### 2.2 Variables From the Medical Records

The following variables from the medical records were retrospectively reviewed and analyzed: patient age, sex, height, weight, compromised host status, fever, dyspnea, type of causative tooth, type of odontogenic infection. Type of odontogenic infection included apical periodontitis, pericoronitis, medication‐related osteonecrosis of the jaw (MRONJ), post‐extraction infection, and osteomyelitis. A compromised host status was defined as positive in a patient with any of the following diseases: rheumatoid arthritis, chronic kidney disease, diabetes, and steroid use.

### 2.3 CT Data Acquisition

CT images were acquired using nine different CT systems including mainly a 64‐slice CT system (Aquilion 64; Canon Medical Systems Corp, Tochigi, Japan) and a 128‐slice CT system (SOMATOM Definition Flash; Siemens, Munich, Germany). Data were acquired under typical head and neck CT scanning conditions (120 kV, 1–5 mm slice) with automatic exposure control. Four different contrast media including mainly Iomeron 300 (Eisai, Tokyo, Japan) and Iopamirdol 370 (Hikari Pharmaceutical, Tokyo, Japan) were used.

### 2.4 Boundaries of the Spaces

The boundaries of each space were defined based on widely accepted definitions: the mylohyoid muscle served as the border between the sublingual and submandibular or submental spaces, and the anterior belly of the digastric muscle marked the boundary between the submental and submandibular spaces.

### 2.5 Abscess Formation and Bone Resorption

**FIGURE 1:**
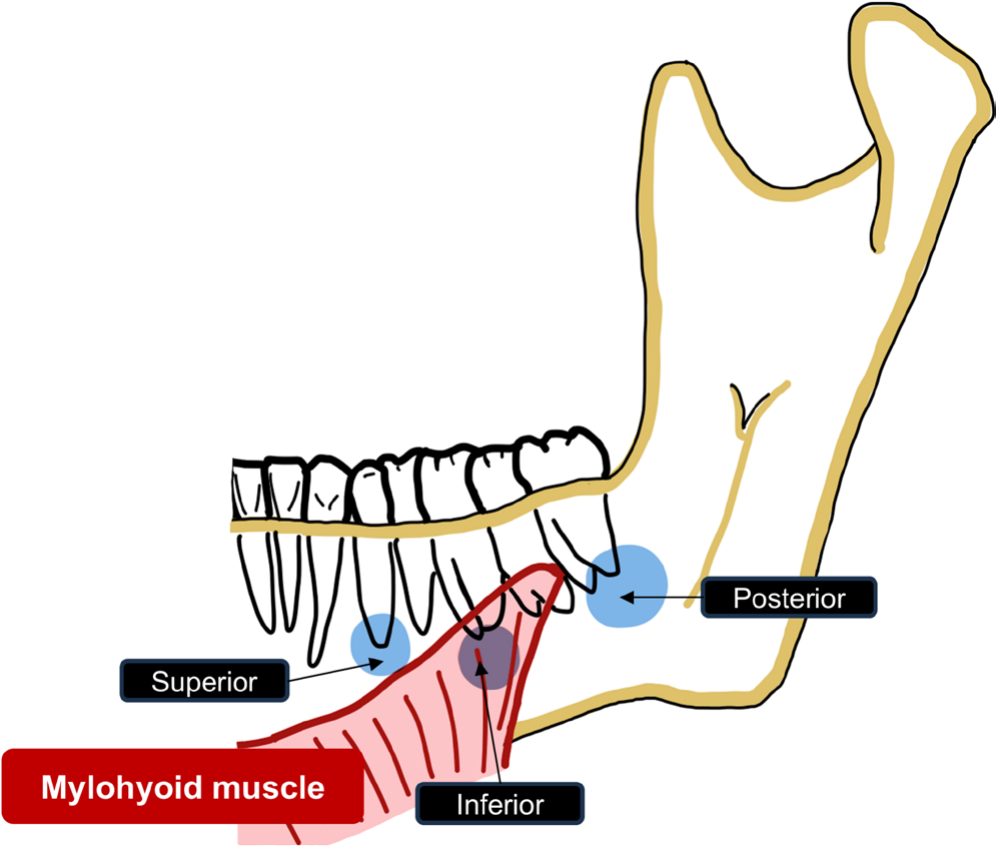
Relationship of the mylohyoid line and bone resorption site. Red line: Mylohyoid line.

The presence of an abscess or gas production was recorded using contrast‐enhanced CT images. Bone resorption site (buccal or lingual side) and its relationship to the mylohyoid line were noted (Figure 1).

### 2.6 Statistical Analysis

All statistical analyses were performed using R version 4.0.5 (R Core Team, Vienna, Austria) and Excel (Microsoft, Redmond, WA, USA). A statistical study was conducted to examine the causes of abscesses in the submental, sublingual, and submandibular spaces as well as the relationship between the mylohyoid line and the spaces. The population was divided into two groups for each of the following three sections. Fisher's exact test was used for nominal variables, Mann–Whitney *U* test for continuous variables, and Bonferroni correction for multiple comparisons. Discriminant analysis was used for multivariate analysis, and a receiver operating characteristic (ROC) curve was used to calculate the cutoff value. The significance level was set at 5%.

### 2.7 Ethics

The study protocol conformed to the ethical guidelines of the Declaration of Helsinki and the Ethical Guidelines for Medical and Health Research involving Human Subjects by the Ministry of Health, Labor, and Welfare of Japan. Ethical approval was obtained from the Institutional Review Board (IRB) (Kurume University Hospital Ethics Review Committee. No. 23122). As this was a retrospective study, identifiable patient information was removed, and the research plan was published on the homepage of each participating hospital's website, along with an opt‐out option in accordance with our IRB instructions. The authors state that every effort was made to follow all local and international ethical guidelines and laws that pertain to the use of human cadaveric donors and their images in anatomical research (Iwanaga et al. 2025; Iwanaga et al. 2022; Iwanaga et al. 2021).

## 3 Results

### 3.1 Patient Characteristics

**FIGURE 2:**
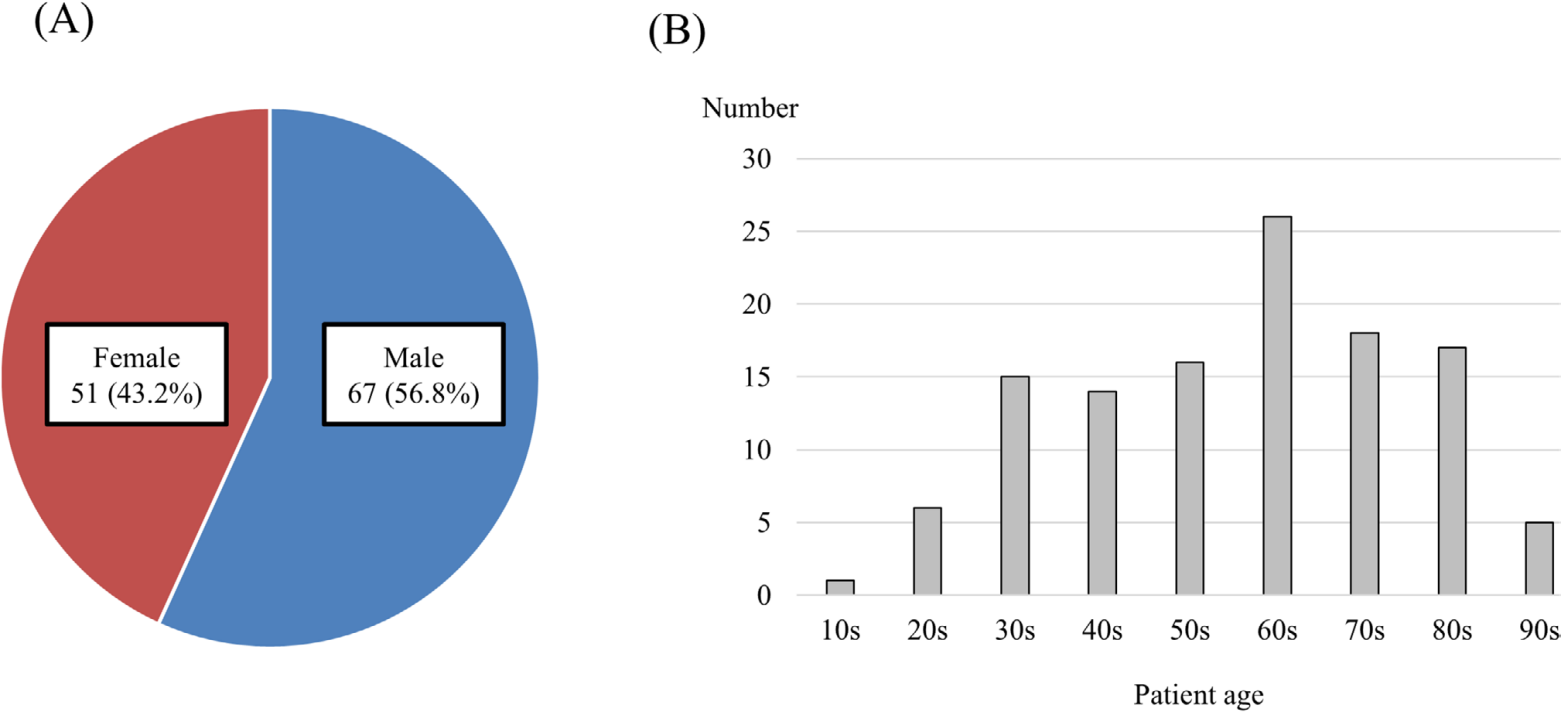
Patient characteristics. (A) Distribution of sex. (B) Distribution of patient age.

**FIGURE 3:**
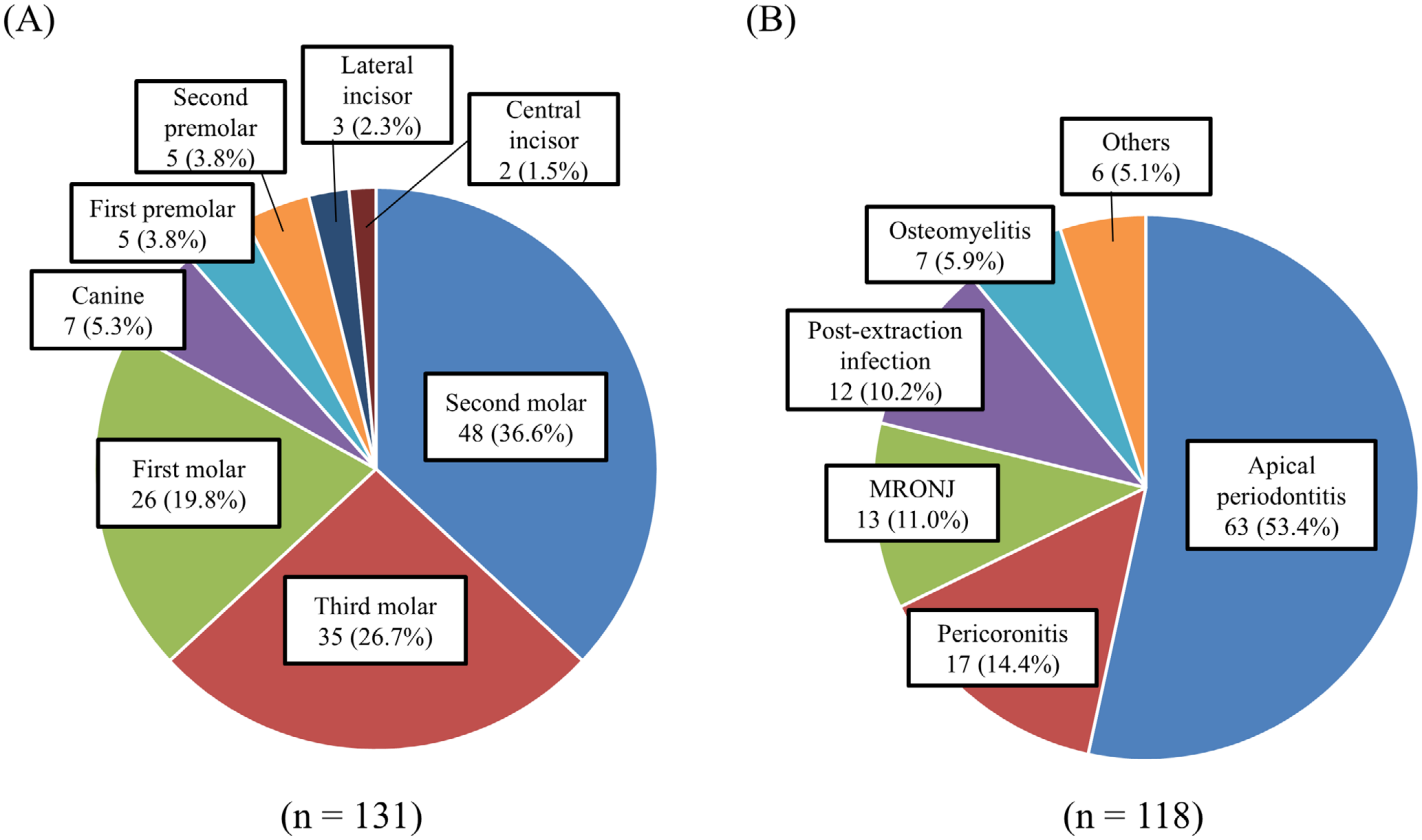
Disease characteristics. (A) Distribution of causative tooth (*n* = 131). (B) Distribution of odontogenic infection (*n* = 118).

One hundred eighteen patients were eligible in this study. Among them, 67 were male (56.8%) and 51 were female (43.2%) (Figure 2A). The most common patient age group was 60's (Figure 2B). The total number of analyzed teeth was 131. The total number of analyzed teeth was 131. Regarding the type of causative tooth, the second molar (36.6%) was the most common, followed by the third molar (26.7%) and the first molar (19.8%) (Figure 3A). Therefore, molars accounted for over 80% of 131 teeth. The breakdown of odontogenic infection was that apical periodontitis was the most frequent, comprising more than half of the total patients (53.4%: 63/118), followed by pericoronitis and MRONJ (Figure 3B). Others (*n* = 6) included periodontitis and radicular cyst (Figure 3B).

A total of 118 patients were classified into three groups based on the location of abscess formation:

Group 1: Patients with abscess in sublingual space alone (*n* = 5).

Group 2: Patients with abscess in “submental or submandibular space” alone (*n* = 90).

Group 3: Patients with abscess in both sublingual space and “submental or submandibular space” (*n* = 23).

### 3.2 Patients With Abscess in Sublingual Space (Group 1 + 3) vs. Patients With Abscess in “Submental or Submandibular Space” Alone (Group 2) (Table 1)

**TABLE 1:** Comparison of patients with abscess in sublingual space (Group 1 + 3) versus patients with abscess in “submental or submandibular space” alone (Group 2).

Variable	Group 1 + 3 (*N* = 28)	Group 2 (*N* = 90)	*p*
*Age (years)*			
Median (range)	52.0 (19–81)	62.7 (21–95)	0.006*
*Sex*			
Male	18 (64.3%)	49 (54.4%)	0.359
Female	10 (35.7%)	41 (45.6%)	
*Height (m)*			
Median (range)	163.3 (142.0–179.6)	159.4 (131.0–185.5)	0.041*
*Weight (kg)*			
Median (range)	62.8 (40.0–82.1)	58.0 (31.5–113.8)	0.040*
*Compromised host*			
Yes	6 (21.4%)	27 (30.0%)	0.377
No	22 (78.6%)	63 (70.0%)	
*Fever (°C)*			
Median (range)	37.5 (36.2–38.9)	37.2 (36.1–39.9)	0.149
*Dyspnea* ^a^			
Yes	11 (39.3%)	27 (30.0%)	0.378
No	17 (60.7%)	62 (68.9%)	
*Type of causative tooth* ^b^			
Anterior	1 (3.6%)	9 (10.0%)	0.577
Premolar	2 (7.2%)	5 (5.6%)	
Molar	25 (89.3%)	81 (90.0%)	
*Bone resorption site* ^c^			
Buccal side	7 (25.0%)	28 (31.1%)	0.418
Lingual side	20 (71.4%)	66 (73.3%)	
No	4 (14.3%)	6 (6.7%)	
*Relationship between mylohyoid line and bone resorption site*			
Superior	12 (42.9%)	9 (10.0%)	0.005*
Inferior	10 (32.1%)	46 (51.1%)	
Posterior	8 (32.1%)	15 (16.7%)	
Other	3 (10.7%)	13 (14.4%)	
*Gas production*			
Yes	1 (3.6%)	13 (14.4%)	0.120
No	27 (96.4%)	77 (85.6%)	

*Note:* A total number in each variable is 118 except for the below. Bold value indicates significant of *P* < 0.05.

^a^ *n* = 117, one patient in Group 2 is excluded due to the lack of data.

^b^ *n* = 123, four patients in Group 2 have multiple causative teeth.

^c^ *n* = 131, 13 patients (three in Group 1 + 3 and 10 in Group 2) have bone resorptions on both sides.

**FIGURE 4:**
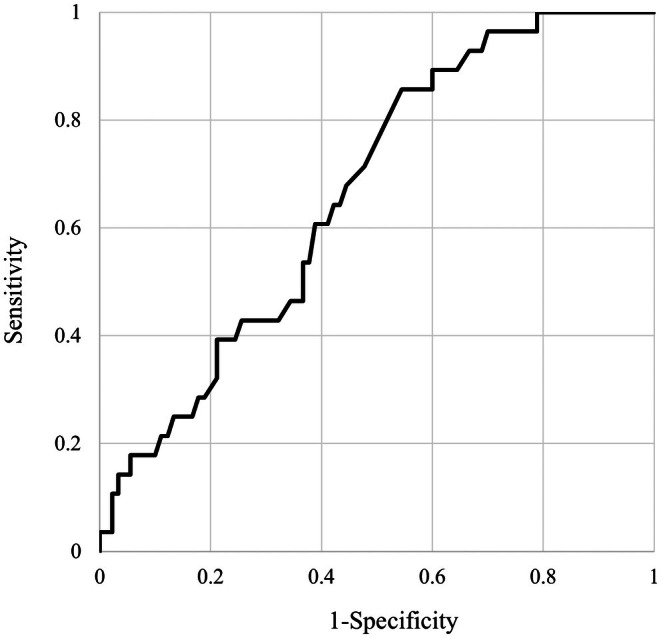
The ROC curve. The ROC curve for accuracy of age in predicting sublingual space abscess. The AUC for our model was 0.666 (95% confidence interval 0.561–0.771).

Significant differences were observed between the two groups in age, height, weight, and the relationship between the mylohyoid line and bone resorption site (Table 1). For age, the cutoff value was decided by using the ROC curve. The score of age ≥ 66.0 years had a sensitivity of 85.7%, a specificity of 45.6%, and area under the curve (AUC) of 0.666 (Figure 4). Multivariate analysis showed that bone resorption superior to the mylohyoid line (odds ratio [OR]: 8.84, 95% confidence interval [CI]: 2.89–27.0) and older than 66.0 years (OR: 0.97, 95% CI: 0.94–1.00) were significantly associated with sublingual space abscess [Table 2].

**TABLE 2:** Multivariate logistic regression analysis concerning the association with sublingual space abscesses.

Variable	*p*	Odds ratio	95% CI	
			Lower	Upper
Superior (vs. Inferior/Posterior/Others)	< 0.001*	8.84	2.87	27.0
Age ≥ 66.0 years (vs. < 66.0 years)	0.028*	0.97	0.94	1.00

*Note:* Bold value indicates significant of *P* < 0.05.

Abbreviation: CI, confidence interval.

### 3.3 In Patients With Submandibular or Submental Space Abscesses (Group 2 + 3), Comparison of Bone Resorption Superior to Mylohyoid Line and Other

Out of 113 patients with submandibular or submental space abscesses (groups 2 + 3), 10 were excluded because the relationship to the mylohyoid line was unclear due to no bone resorption. Of the remaining 103 patients, 19 patients had bone resorption superior to the mylohyoid line (Group A), while the other 84 patients had bone loss in other areas (Group B) (Table 3). The presence of dyspnea, and the presence of sub‐masseteric space abscess had statistically significant differences between the two groups (Table 3). Multivariate analysis showed that the presence of sublingual space abscess (OR: 16.8, 95% CI: 3.96–71.6) and dyspnea (OR: 0.16, 95% CI: 0.03–0.85) were significantly associated with bone resorption superior to the mylohyoid line (Table 4).

**TABLE 3:** Comparison of bone resorption superior to mylohyoid line (Group A) and other regions (Group B) in patients with submandibular or submental space abscesses (Group 2 + 3).

Variable	Group A (*N* = 19)	Group B (*N* = 84)	*p*
*Age (years)*			
Median (range)	60.3 (19–93)	60.3 (23–92)	0.990
*Sex*			
Male	13 (68.4%)	47 (56.0%)	0.320
Female	6 (31.6%)	37 (44.0%)	
*Height (m)*			
Median (range)	163.9 (137.0–185.5)	158.4 (131.0–179.6)	0.061
*Weight (kg)*			
Median (range)	63.0 (46.5–90.8)	58.5 (31.5–113.8)	0.076
*Compromised host*			
Yes	4 (21.1%)	23 (27.4%)	0.571
No	15 (78.9%)	61 (72.6%)	
*Fever (°C)*			
Median (range)	37.4 (36.2–38.8)	37.2 (36.1–39.9)	0.192
*Dyspnea* ^a^			
Yes	3 (15.8%)	33 (39.3%)	0.049*
No	16 (84.2%)	50 (60.7%)	
*Type of causative tooth* ^b^			
Anterior	2 (10.5%)	5 (6.0%)	0.739
Premolar	1 (5.3%)	4 (4.8%)	
Molar	16 (84.2%)	78 (92.9%)	
*Bone resorption site* ^c^			
Buccal side	11 (57.9%)	21 (25.0%)	0.039*
Lingual side	13 (68.4%)	71 (84.5%)	
*Deep neck space with abscess*			
Sublingual space	10 (52.6%)	10 (11.9%)	< 0.001*
Parotid space	0 (0.0%)	7 (8.3%)	0.192
Temporal fossa/infratemporal fossa	1 (5.3%)	7 (8.3%)	0.652
Pteryngomandibular space	5 (26.3%)	17 (20.2%)	0.559
Submasseteric space	1 (5.3%)	22 (26.2%)	0.048*
Parapharyngeal space	2 (10.5%)	12 (14.3%)	0.666
Retropharyngeal space	2 (10.5%)	2 (2.4%)	0.097
*Gas production*			
Yes	2 (10.5%)	10 (11.9%)	0.866
No	17 (89.5%)	74 (88.1%)	

*Note:* Out of 113 patients in Group 2 + 3, 10 patients are excluded due to lack of data on the relationship between the mylohyoid line and bone resorption. A total number in each variable is 103 except for the below. Bold value indicates significant of *P* < 0.05.

^a^ *n* = 102, one patient in Group B was excluded due to the lack of data.

^b^ *n* = 106, three patients in Group B had multiple causative teeth.

^c^ *n* = 116, 13 patients (five in Group A and eight in Group B) had bone resorptions on both sides.

**TABLE 4:** Multivariate logistic regression analysis concerning the association with superior group.

Variable	*p*	Odds ratio	95% CI	
			Lower	Upper
Sublingual space abscess (vs. Absence)	< 0.001*	16.8	3.96	71.6
Dyspnea (vs. Absence)	0.032*	0.16	0.03	0.85

*Note:* Bold value indicates significant of *P* < 0.05.

Abbreviation: CI, confidence interval.

### 3.4 Patients With Sublingual Space Abscess Alone (Group 1) vs. Patients With Sublingual Space Abscess and “Submandibular or Submental” Space Abscess (Group 3)

**FIGURE 5:**
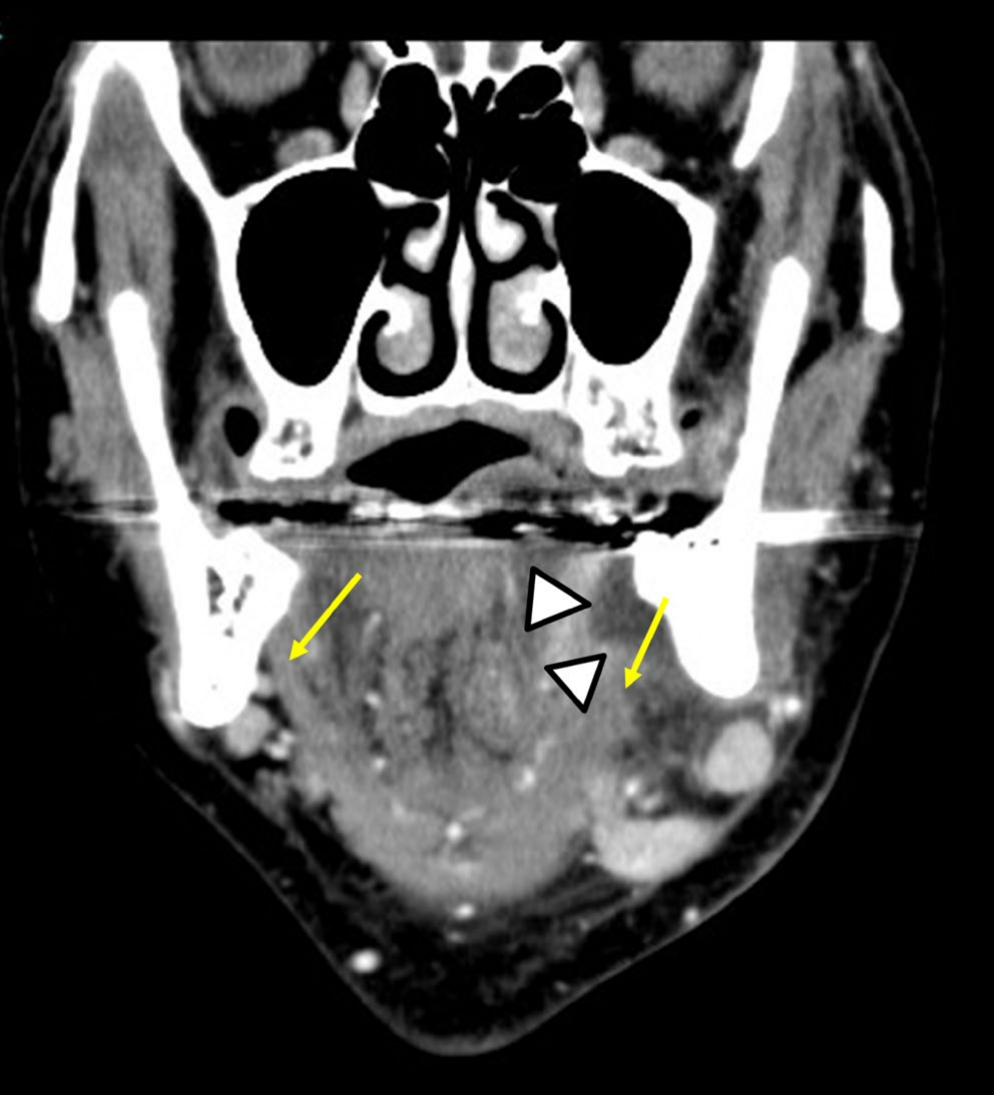
Coronal contrast‐enhanced CT image of a patient with sublingual space abscess alone (Group 1). White arrow: Abscess. Yellow arrow: Mylohyoid muscle.

Five patients had a sublingual space abscess alone (Group 1), while 23 patients had both sublingual and “submandibular or submental” space abscesses (Group 3) (Table 5). Among five patients in Group 1, the types of causative teeth were as follows: one second pre‐molar, one first molar, two second molars, and one third molar [Table 6]. The relationship between the mylohyoid line and the bone resorption site in those teeth varied. For instance, one case was superior to the line and one was inferior, even for the same second molar (Table 6). There were no significant factors identifying them in abscess formation in the sublingual space and “submandibular or submental” space [Table 5]. We present a case of a sublingual space abscess alone (Figure 5). This case corresponds to patient number 2 in Table 6.

**TABLE 5:** Comparison of patients with sublingual space abscess alone (Group 1) and patients with sublingual space abscess and “submandibular or submental” space abscess (Group 3).

Variable	Group 1 (*N* = 5)	Group 3 (*N* = 23)	*p*
*Age (years)*			
Median (range)	48.0 (41–81)	62.7 (21–95)	0.749
*Sex*			
Male	3 (60.0%)	15 (65.2%)	1.000
Female	2 (40.0%)	8 (34.8%)	
*Height (m)*			
Median (range)	164.9 (146.0–179.6)	163.8 (142.0–179.6)	0.499
*Weight (kg)*			
Median (range)	60.2 (43.0–82.1)	63.3 (40.0–82.1)	0.155
*Compromised host*			
Yes	0 (0.0%)	7 (30.4%)	0.290
No	5 (100.0%)	16 (69.6%)	
*Fever (°C)*			
Median (range)	37.9 (36.4–38.8)	37.4 (36.2–38.8)	0.477
*Dyspnea*			
Yes	1 (20.0%)	10 (43.5%)	0.620
No	4 (80.0%)	13 (56.5%)	
*Type of causative tooth*			
Anterior	0 (0.0%)	1 (4.3%)	0.431
Premolar	1 (20.0%)	1 (4.3%)	
Molar	4 (80.0%)	21 (91.4%)	
*Bone resorption site* ^a^			
Buccal side	1 (20.0%)	6 (26.1%)	0.604
Lingual side	4 (80.0%)	16 (69.6%)	
No	0 (40.0%)	4 (17.4%)	
*Relationship between mylohyoid line and bone resorption site*			
Superior	2 (40.0%)	10 (43.5%)	0.559
Inferior	3 (60.0%)	7 (30.4%)	
Posterior	0 (0.0%)	0 (0.0%)	
Other	0 (0.0%)	2 (8.7%)	
*Gas production*			
Yes	0 (0.0%)	1 (4.3%)	1.000
No	5 (100.0%)	22 (95.7%)	

*Note:* A total number in each variable is 28 except for the below.

^a^ *n* = 31, three patients in Group 3 have bone resorptions on both sides.

**TABLE 6:** Breakdown of group 1 patients.

Patient no.	Age	Sex	Causative tooth	Relationship between mylohyoid line and bone resorption site
1	48	Male	Second molar	Superior
2	66	Female	First molar	Superior
3	64	Female	Second premolar	Inferior
4	41	Male	Second molar	Inferior
5	24	Male	Third molar	Inferior

## 4 Discussion

This study recruited 118 patients with mandibular odontogenic deep neck abscesses in a multicenter setting and analyzed their transmission pathways focusing on the mylohyoid line. We found that bone resorption superior to the mylohyoid line and the related presence of abscess in the submandibular or submental space were both significantly associated with the presence of sublingual space abscess. In addition, the type of causative tooth (i.e., anterior, premolar, or molar) was not a significant risk factor in any of the three analyses: (1) abscess formation in the sublingual space, (2) abscess formation in the submandibular or submental space associated with bone resorption superior to the mylohyoid line, and (3) abscess formation in both the sublingual and “submandibular or submental” spaces.

### 4.1 Abscess Formation and Advanced Age

Sublingual space abscess was associated with advanced age (OR: 0.97), the odontogenic lesion superior to the mylohyoid line (OR: 8.84), and submandibular or submental space abscess due to the odontogenic lesion superior to the mylohyoid line (OR: 16.8). Advanced age is associated with sublingual space abscesses, which may progress to involve the submandibular or submental spaces. This finding supports previous studies identifying advanced age as a risk factor for the severity of DNIs (Iwata et al. 2021; Chen et al. 2021).

### 4.2 Hiatus in the Mylohyoid Muscle as a Pathway for Infection Spread

**FIGURE 6:**
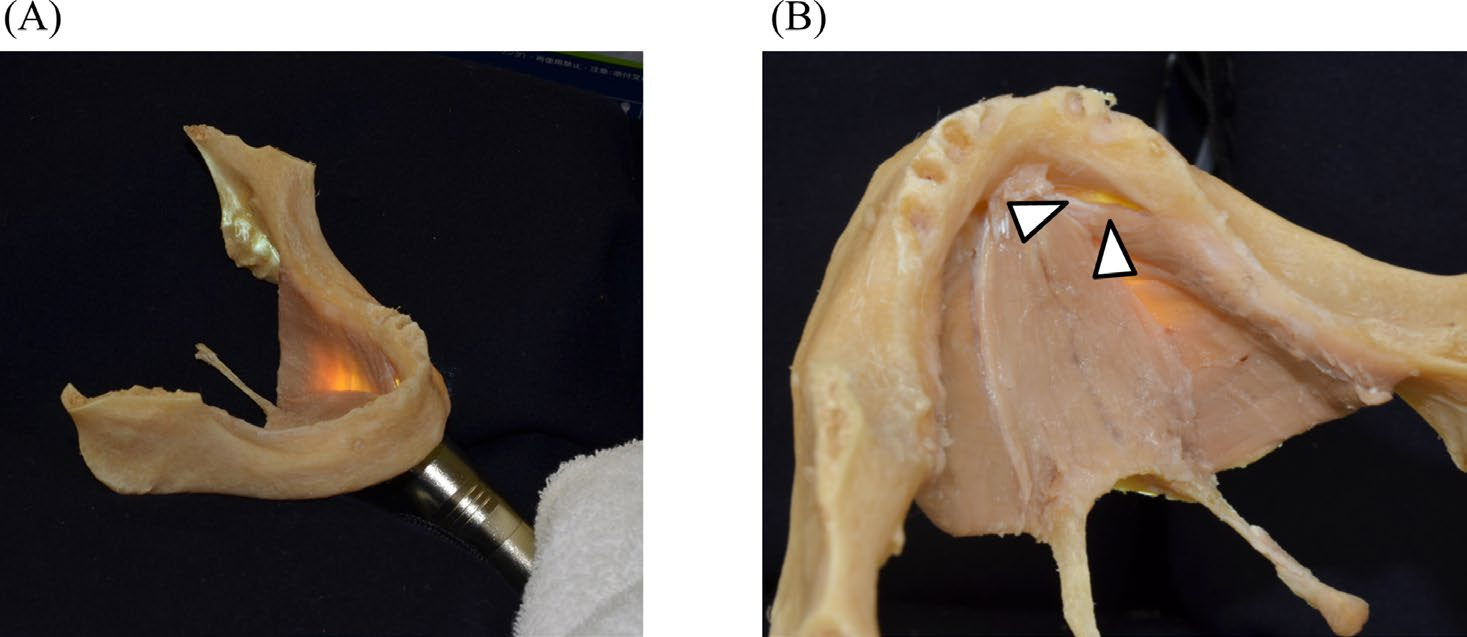
Mylohyoid muscle. (A) This image presents an inferior view of the mylohyoid muscle, illuminated from below to enhance visualization of its fiber orientation and structural contours. The muscle forms a sling‐like diaphragm that constitutes the floor of the oral cavity, extending from the mandible to the hyoid bone. (B) A close‐up view highlights a natural anatomical gap—referred to as the mylohyoid hiatus—located in the anterior one‐third of the muscle. This hiatus represents a separation between muscle bundles, visible as a translucent area (see arrow), and serves as a potential conduit for the spread of infection from the sublingual space to adjacent compartments such as the submandibular and submental spaces.

**FIGURE 7:**
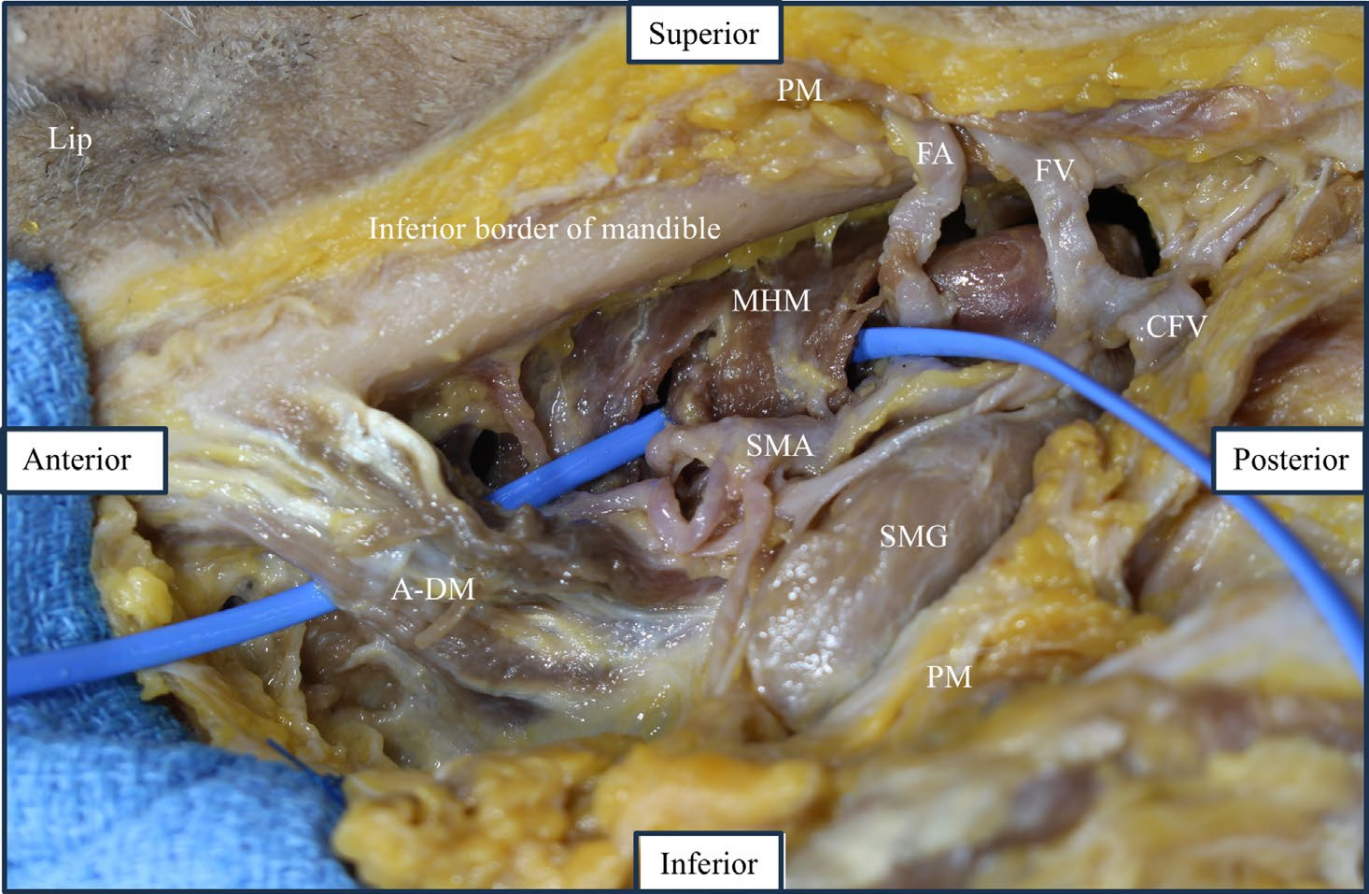
Floor of the mouth and inflammatory spread pathway. This anatomical illustration depicts the floor of the mouth and surrounding structures, emphasizing the potential route of inflammatory spread in odontogenic infections. A blue string has been placed to simulate the path of infection transmission from the sublingual space through the mylohyoid hiatus and posterior margin of the mylohyoid muscle into the submandibular and submental spaces. A‐DM: Anterior belly of digastric muscle. PM: Platysma. SMG: Submandibular grand; SMA: Submental artery. MHM: Mylohyoid muscle. FA: Facial artery. FV: Facial vein. CFV: Central facial vein. This image, obtained from the Tulane Clinical Anatomy Research Lab, visually supports the hypothesis that anatomical discontinuities in the mylohyoid muscle may facilitate the downward spread of odontogenic infections, particularly when bone resorption occurs superior to the mylohyoid line.

A hiatus in the mylohyoid muscle, ranging from 42.0% to 77.0%, allows penetration of the sublingual gland, adipose tissue, and the submental vessels and their branches (Yang et al. 2016; Windisch et al. 2004). It is reported that the anterior one‐third of the mylohyoid muscle is responsible for over half of the hiatus (Figure 6), while the posterior one‐third contributes to it in less than 10% of the population (Obata et al. 2024). The hiatus, along with the gap between the posterior border of the mylohyoid muscle and the hyoglossus muscle, called the “glosso‐mylohyoid gap,” is considered a potential pathway for the spread of inflammation from the sublingual space to the submandibular or submental space (Otonari‐Yamamoto et al. 2010) (Figure 7). This is the same pathway for the spread of plunging ranula to the submandibular or submental space, which originates from the sublingual gland located superior to the mylohyoid muscle (Otonari‐Yamamoto et al. 2010).

### 4.3 Bone Resorption Superior to the Mylohyoid Line

We found that bone resorption superior to the mylohyoid line and the associated presence of abscess in the submandibular or submental space were both significantly associated with the presence of sublingual space abscess. Besides, dyspnea was also significantly related to abscess formation in the submandibular or submental space when associated with bone resorption superior to the mylohyoid line (OR: 0.16). In other words, the proportion of patients in Group A (superior) who complained of dyspnea was significantly lower than those in Group B (other). This is thought to be due to the anatomical distance from the upper airway—lesions superior to the mylohyoid line are generally more anterior and less likely to cause airway compression than those located inferior or posterior.

The significant association between bone resorption superior to the mylohyoid line and sublingual space abscess formation may be explained by anatomical features of the mylohyoid muscle and surrounding fascial planes. Although the mylohyoid muscle forms the floor of the oral cavity, it is not a complete barrier; it contains natural hiatuses, such as the posterior mylohyoid gap, through which infection can pass. This anatomical opening allows direct communication between the sublingual and submandibular spaces, facilitating the spread of infection from superior regions into deeper neck compartments. Furthermore, the continuity of cervical fascial planes—especially the superficial and deep layers—may promote multidirectional transmission of odontogenic infections. Anatomical variability in the position and thickness of the mylohyoid muscle, as well as the location of tooth apices relative to the mylohyoid line, may also contribute to differences in infection pathways among patients. These factors should be considered when evaluating the risk and direction of abscess formation in clinical settings.

### 4.4 Causative Teeth and Mylohyoid Line

Previous reviews described that inflammation caused by premolars tends to spread to the sublingual space and that caused by molars, especially the second and third molars tends to spread to the submandibular space (Patel and Bhatt 2018; French et al. 2017). In this study, the causative teeth in patients with sublingual space abscess alone (Group 1) were as follows: one second premolar, two first molars, and two second molars. Those reviews noted the reason for the above‐mentioned that the apex of mandibular premolars is often located superior to the mylohyoid line and those of second and third molars are generally located inferior to the mylohyoid line (Patel and Bhatt 2018; French et al. 2017). In the present study, the relationship between the mylohyoid line and the bone resorption site in causative teeth varied. For instance, with one case being superior to the line and one being inferior, even for the same second molar. In addition, the type of causative tooth (i.e., anterior, premolar, or molar) was not a significant risk factor in any of the three analyses: (1) abscess formation in the sublingual space, (2) abscess formation in the submandibular or submental space associated with bone resorption superior to the mylohyoid line, and (3) abscess formation in both the sublingual and “submandibular or submental” spaces. These results indicate that the primary factor associated with the transmission pathway is not the type of causative tooth but the relationship between the mylohyoid line and the bone resorption site in those teeth.

### 4.5 Airway Obstruction

Airway obstruction is a critical and potentially life‐threatening complication in patients with odontogenic DNIs, requiring prompt evaluation and intervention (Cho et al. 2016). In this study, we found that cases with bone resorption superior to the mylohyoid line exhibited significantly fewer instances of dyspnea compared to those with bone resorption located posterior or inferior to this anatomical landmark. This finding can be explained by considering the anatomical pathways and the potential for infection spread. Infections located below the mylohyoid muscle are more prone to rapid expansion and may lead to airway compromise through mechanisms such as tongue base edema or posterior pharyngeal wall bulging. In particular, infections involving the retropharyngeal spaces can cause posterior displacement of the airway, exacerbating respiratory difficulty. In this regard, we previously proposed that tracheostomy is preferable for patients with odontogenic retropharyngeal space abscesses (Iwata et al. 2025). Our findings highlight the importance of lesion localization in the initial assessment of odontogenic deep neck infections.

### 4.6 Consideration of Steroid Therapy

In the management of odontogenic DNIs, the use of corticosteroids remains a topic of clinical debate. While antibiotics and surgical drainage are the mainstays of treatment, adjunctive steroid therapy may offer benefits in select cases, particularly those at risk of airway compromise (Kent et al. 2021). Corticosteroids exert potent anti‐inflammatory effects that can reduce tissue edema, especially in the tongue base, pharyngeal walls, and surrounding soft tissues (Kent et al. 2021). This reduction in swelling may alleviate airway obstruction and improve respiratory symptoms, potentially delaying or even avoiding the need for invasive airway management (Reichardt et al. 2021). Our results may suggest that cases with lesions located below the mylohyoid muscle—where the risk of airway compromise is higher—may particularly benefit from early steroid administration to mitigate inflammation‐induced narrowing of the airway. However, the use of steroids must be carefully balanced against potential risks. Immunosuppression may impair host defense mechanisms, particularly in the presence of uncontrolled infection or abscess formation (Goldmann et al. 2024). Therefore, close monitoring is essential to ensure that steroid therapy does not mask worsening infection or delay necessary interventions. Recent studies suggest that short‐term, low‐to‐moderate dose corticosteroids may be safe and effective in reducing airway edema and improving clinical outcomes in DNIs, especially when used judiciously in conjunction with definitive treatment (Bandol et al. 2025). Prospective studies are needed to establish standardized protocols and identify patient populations most likely to benefit from steroid therapy.

To our knowledge, previous studies examined the spread of odontogenic infections, but they primarily focused on the diagnostic value of CT and the characteristics of severe cases (Wabik et al. 2014; Rautaporras et al. 2023). It is generally accepted that the infections spread to the submandibular space by passing around the posterior edge of the mylohyoid muscle (Patel and Bhatt 2018; French et al. 2017), and from there to the submental space by tracking around the anterior belly of the digastric muscle (Patel and Bhatt 2018; French et al. 2017). The hiatus could be an alternative pathway. However, there has been no prior evidence‐based discussion regarding the frequency of each transmission pathway—the mylohyoid hiatus and the posterior border of the mylohyoid muscle.

In conclusion, this is the first evidence to indicate that the mylohyoid muscle affects the route of spread of mandibular odontogenic deep neck infection. Our results suggest that when the lesion is located superior to the mylohyoid line, an abscess forms in the sublingual space, and then progresses to the submandibular or submental space. In addition, the type of causative tooth (i.e., anterior, premolar, or molar) was not a significant risk factor in any of the three analyses: (1) abscess formation in the sublingual space, (2) abscess formation in the submandibular or submental space associated with bone resorption superior to the mylohyoid line, and (3) abscess formation in both the sublingual and “submandibular or submental” spaces. In other words, since any tooth can cause an abscess to form not only in the sublingual space but also in the submandibular or submental space, oral and maxillofacial surgeons should carefully identify the causative tooth.

## 5 Limitations

Given the tendency of deep neck abscesses to spread to adjacent anatomical spaces, cases with abscesses confined to a single space are relatively uncommon. In this study, only five patients presented with an isolated sublingual space abscess. Consequently, we cannot exclude the possibility that involvement of adjacent deep neck spaces may have influenced the clinical presentation and CT findings. This limitation should be considered when interpreting the results. Furthermore, as a retrospective multicenter study, our findings are subject to selection bias and limited control over confounding variables. The relatively small number of patients with isolated sublingual space abscesses also limits the statistical power of subgroup analyses. Therefore, caution is warranted to avoid overinterpretation of these results. Future prospective studies with larger and more diverse populations are needed to validate our findings and clarify the anatomical and clinical determinants of abscess spread. Our data suggest that molars—particularly second and third molars—were the most frequent causative teeth, accounting for over 80% of cases. Age also appeared to influence abscess location, with patients aged ≥ 66.0 years more likely to develop sublingual space abscesses. Although gender differences were not statistically significant in this cohort, further investigation may be warranted. These observations underscore the importance of considering individual anatomical and demographic factors when evaluating and managing mandibular odontogenic DNIs.

## Author Contributions

**Eiji Iwata:** conceptualization, investigation, writing – original draft, data curation, formal analysis, writing – review and editing, project administration. **Kyoichi Obata:** conceptualization, investigation, formal analysis. **Shogo Kikuta:** conceptualization, investigation, formal analysis, writing – original draft. **Naoki Kaneko:** conceptualization, investigation. **Kotaro Sato:** conceptualization, investigation. **Norio Kitagawa:** conceptualization. **Yohei Takeshita:** conceptualization. **Katsuhisa Matsuo:** investigation. **Junsei Sameshima:** investigation. **Akira Tachibana:** supervision. **Shintaro Kawano:** supervision. **Jingo Kusukawa:** supervision. **Masaya Akashi:** supervision. **Soichiro Ibaragi:** supervision. **Joe Iwanaga:** conceptualization, writing – original draft, writing – review and editing.

## Conflicts of Interest

The authors declare no conflicts of interest.

## Data Availability

The data that support the findings of this study are available from the corresponding author upon reasonable request.
